# Comparing GLP-1 agonists versus other weight loss interventions on risk of atrial fibrillation recurrence after catheter ablation: a meta-analysis

**DOI:** 10.1007/s10840-026-02297-8

**Published:** 2026-03-17

**Authors:** Hoi-Ying Li, Gregory Y. H. Lip, Tsz-Kwan Chan, Kai-Hang Yiu, Hung-Fat Tse, Claire A. Martin, Yap-Hang Chan

**Affiliations:** 1 Department of Medicine, School of Clinical Medicine, The University of Hong Kong, Queen Mary Hospital, Office K1927C, 102 Pokfulam Road, Hong Kong SAR, China; 2 Department of Cardiology, Royal Papworth Hospital, Cambridge Biomedical Campus, Cambridge, UK; 3 Visiting Fellowship, Hughes Hall, University of Cambridge, Cambridge, UK; 4 Division of Experimental Medicine and Immunotherapeutics, Cambridge University Hospitals NHS Foundation Trust/University of Cambridge, Cambridge, UK; 5 Liverpool Centre for Cardiovascular Science at University of Liverpool, Liverpool John Moores University and Liverpool Heart and Chest Hospital, Liverpool, UK; 6 Department of Clinical Medicine, Aalborg University, Aalborg, Denmark; 7 Department of Medicine, Shenzhen Hong Kong University Hospital, Shenzhen, China; 8 Institute of Cardiovascular Science and Medicine, The University of Hong Kong, Hong Kong SAR, China; 9 Department of Clinical Medicine, University of Cambridge, Cambridge, UK

**Keywords:** GLP-1 agonist, Weight reduction, Atrial fibrillation, Obesity, Catheter ablation, Meta-analysis

## Abstract

**Background:**

Obesity often co-exists with Atrial Fibrillation (AF) and poses challenges to ablation success. Whether glucagon-like peptide-1 (GLP-1) agonists have a role and how they compare to other weight loss interventions in preventing AF recurrence after catheter ablation remains unclear.

**Methods:**

We performed meta-analysis and included prior interventional and observational studies that investigated weight loss-related interventions on AF recurrence post-ablation. Included were weight loss interventions that entailed perioperative use of GLP-1 agonists (4 studies), non-pharmacological interventions such as lifestyle/ risk factors modifications (8 studies), and bariatric surgery (1 study). Primary outcome was recurrence of clinical AF or a requirement for further rhythm control post-ablation.

**Results:**

Two randomized-control trials and 11 cohorts were included (*n* = 5,415). Overall, weight loss interventions were associated with significant reduction in AF recurrence post-ablation (*RR* = 0.63 [95% CI: 0.46—0.87]). Bariatric surgery was associated with reduced risk of AF recurrence (*RR* = 0.31 [0.13 to 0.75]), while GLP-1 agonists and lifestyle /risk factors modifications showed trends towards protection with borderline statistical significance (*RR* = 0.64 [95% CI 0.36 to 1.14]). However, only 1 study investigated bariatric surgery which should be interpreted with caution. Importantly, meta-regression showed that for each 1% absolute decrease in body weight achieved, there was 6.3% relative-risk reduction in AF recurrence (*RR* = 0.94 [0.90—0.98]), regardless of weight loss intervention subtype. Sustained continuation of weight loss post-ablation was a determining factor of lower recurrence (*P* = 0.03).

**Conclusions:**

Peri-operative weight loss interventions in obesity patients significantly reduced AF recurrence post-ablation, in a dose–response manner and irrespective of choice of intervention strategy. Sustained continuation of weight loss post-ablation is a key determinant of success.

**Graphical abstract:**

Forest plot for recurrence of AF stratified by interventions. Overall pooled risk ratio (RR) across all weight-loss interventions was 0.64 (95% CI: 0.47–0.87, heterogeneity: *I*² = 86 & τ2 = 0.23).

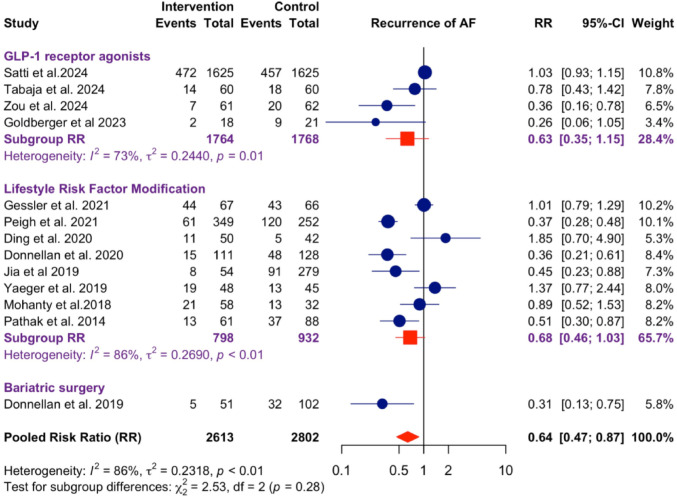

**Supplementary Information:**

The online version contains supplementary material available at 10.1007/s10840-026-02297-8.

## Key points

In this meta-analysis studying 5,415 patients that include 2 randomized controlled trials, peri-operative weight loss interventions significantly reduced AF recurrence after catheter ablation, irrespective of the choice of intervention strategy.

Meta-regression shows that for each 1% absolute decrease in body weight achieved, there was 6.3% relative risk reduction in AF recurrence.

Sustained continuation of weight loss beyond ablation was a determining factor of lower AF recurrence.

**Supplementary Information:**

The online version contains supplementary material available at 10.1007/s10840-026-02297-8.

## Take home message

Weight loss in obesity patients significantly reduced AF recurrence after an ablation procedure, in a dose–response manner and irrespective of the choice of intervention strategy. Sustained weight control is a key determinant of success.

**Supplementary Information:**

The online version contains supplementary material available at 10.1007/s10840-026-02297-8.

## Background

Atrial fibrillation (AF) is the most common sustained cardiac arrhythmia, with a rising incidence and prevalence globally [1]. Obesity significantly increases the risk of AF through a multitude of mechanisms that alter cardiac structure and function, and modulate indirect hemodynamic, metabolic, and inflammatory predispositions [2, 3]. Catheter ablation is an effective therapeutic strategy for arrhythmia control in AF, especially at an early stage [1]. Nevertheless, obesity reduces procedural success and increases the risk of AF recurrence after ablation [2]. Therefore, weight loss has been recognized as an important element in clinical management strategies to improve rhythm control in AF in persons with obesity [1] [3].

Long-term sustained weight loss has been associated with significant reductions of AF burden and facilitates the maintenance of sinus rhythm [4]. Glucagon-like peptide-1 receptor agonists (GLP-1 RA), first used to treat type 2 diabetes, contribute to weight loss [5] through suppressing signals from neurons promoting hunger and enhance signals associated with fullness [6, 7]. GLP-1 RA are a potential therapeutic option for obese patients undergoing AF ablation due to their weight loss effect and potential cardiovascular and metabolic benefits [5]. However, the effectiveness of GLP-1 RA in reducing AF recurrence post-ablation compared to other weight loss interventions, such as lifestyle risk factor modification (LRFM) and bariatric surgery, remains uncertain.

We therefore performed this meta-analysis to primarily investigate the comparative effects of GLP-1 RA versus other weight loss interventions on AF recurrence following catheter ablation in obese patients.

## Methods

### Search strategy and study selection

A systematic review and meta-analysis have been performed according to the PRISMA guidelines [8] to investigate the effect of GLP-1 RA versus other weight loss interventions on the risk of AF recurrence after ablation in overweight or obese patients. “Exercise”, “Diet”, “Risk factor modification”, “Lifestyle management”, “Weight loss”, “Overweight”, “Obesity”, “Obese”, “Surgery”, “Glucagon-like peptide 1 receptor agonists”, “Exenatide”, “Lixisenatide”, “Liraglutide”, “Dulaglutide”, “Semaglutide”, “Albiglutide”, “Efpeglenatide” and “Atrial fibrillation ablation” were used as the keywords in the search process. There were no restrictions on publication year. Studies published in English, French, German, Italian, Spanish, and Chinese languages were included. Eligible studies involved patients using GLP-1 RA or patients who were overweight or obese and received other perioperative weight loss interventions before or after AF ablation, compared with a control group receiving standard treatment or no weight loss intervention. We included randomised controlled trials (RCTs) and cohort studies in the analysis, excluding case reports, reviews, and cross-sectional studies. The primary outcome was AF recurrence post-ablation with different weight loss interventions, with the secondary outcome being the timing of weight loss intervention.

The literature search was performed on PubMed, Scopus, Embase, and Cochrane Library, with the last search conducted on 09 Oct 2024. An additional search was conducted on Heart Rhythm to include relevant conference abstracts. All search was performed twice by two independently investigators (H.Y.L. and T.K.C.), and further verified by a third senior investigator (Y.H.C.). All included studies were reviewed independently and assessed for quality. STROBE checklist [9] was used for the quality assessment of cohort studies, while the CONSORT checklist [10] was used to assess the quality of randomised controlled trials (RCTs). Studies that met less than 50% of the checklist items would be excluded from the analysis to avoid the inclusion of poor-quality studies.

### Ethics Approval

As this is a meta-analysis of published studies, there is no direct patient clinical contact. Therefore, no specific ethical approval was obtained or deemed necessary.

### Data extraction

Data regarding AF recurrence 12 months post-ablation, methods of weight-loss intervention and initial and completion time of interventions were extracted from each study. Weight loss interventions were grouped into three categories: GLP-1 receptor agonists (GLP-1 RA), Lifestyle risk factor modification (LRFM), and bariatric surgery. GLP-1 RA involves the perioperative use of medications such as Semaglutide, Liraglutide, and other agents that target glucagon-like peptide-1 receptors to facilitate weight loss [11–14]. LRFM focuses on promoting a healthy lifestyle and managing cardiovascular disease (CVD) risk factors, including hypertension, diabetes, and sleep apnoea. Studies in this category implemented various strategies, such as intermittent fasting, lifestyle counselling, physical training, and professional-led risk factor modification clinics to support participants in achieving weight loss [15–21]. In one study (Jia et al.) [22], the weight loss intervention used was not specified. It is assumed that patients at least partially adopted lifestyle modifications to achieve weight loss. Bariatric surgery was defined as a surgical weight loss method, particularly used in morbidly obese patients, that was conducted pre-ablation to reduce weight and improve comorbidities prior to ablation [23].

For studies with follow-up periods that did not correspond to 12 months, recurrence data closest to this duration were collected to ensure more accurate comparisons between studies. Additionally, baseline characteristics and BMI at the end of the follow-up period were also collected and summarised.

### Definition of primary outcome

The primary outcome was defined as recurrence of clinical AF, documented AF or atrial arrhythmia with a duration ≥ 30 s, or a requirement for further rhythm control post-ablation including new initiation of class I/III anti-arrhythmic drug therapies, cardioversion, or further ablation.

### Statistical analysis

All analyses used the “meta” package in R Studio version 2024. A random-effects model using the Inverse Variance method was conducted to derive risk ratio (RR) with 95% confidence intervals (CI) estimates of AF recurrence. Between-study variance (τ^2^) was estimated using the Restricted Maximum Likelihood (REML) method to account for heterogeneity. Heterogeneity between studies was evaluated using the I^2^ statistic and τ^2^, with *I*^2^ > 50% and τ^2^ > 0.1 suggesting a significant heterogeneity. Forest plots were drawn to visualise the effects of each outcome.

Subgroup analysis was done using a Chi-squared test for group differences to assess the effects on the types and time of weight-loss interventions. Meta-regression analyses were also conducted to assess factors that affect the heterogeneity in outcomes between studies. Sensitivity analysis was conducted using the leave-one-out method and exclusion of conference abstracts to assess the influence of the individual study. Publication bias was assessed using funnel plots and Egger’s test.

## Results

A total of 3092 records resulted after medical database searching. After removing 880 duplicates, 2212 studies underwent title and abstract screening, and 2193 studies were removed. Following a full-text screening of the remaining 19 studies, 2 studies were excluded due to overlapping cohorts and 4 studies were excluded as irrelevant. Finally, 11 cohort studies (including 2 conference abstracts) and 2 RCTs (including 1 conference abstract) published between 2014–2024 were included in the meta-analysis (Supplementary Fig. 1). All included studies were of high quality and aligned with at least 80% of the criteria outlined in the quality assessment checklist. A summary of the included studies was concluded in Table 1. A total of 2613 patients in the intervention group and 2802 patients in the control group across 12 studies in English and 1 in Chinese were included in the analysis. The baseline characteristics and the definition of the recurrence of AF in each study were summarised in Supplementary Tables 1 and 2.

**Table 1:** Summary of included studies

Study	Type of study	Country	Weight loss intervention	Initial time of intervention	Duration of intervention
Satti et al.2024	Retrospective matched cohort	US	Various GLP-1 receptor agonists	Within 1 year pre-ablation	Not Specified
Tabaja et al. 2024*	Retrospective matched cohort	US	Various GLP-1 receptor agonists	Within 3 months pre or post ablation	Ongoing treatment longer than 6 months
Zou et al. 2024*	Retrospective matched cohort	US	Semaglutide	Not Specified	Not Specified
Goldberger et al. 2023*	Randomized Controlled Trials	US	Liraglutide	3 months pre-ablation	Ongoing treatment
Gessler et al. 2021	Randomized Controlled Trials	Germany	Nutrition advice and physical training	Post-ablation	12 months
Peigh et al. 2021	Retrospective cohort	US	Healthy lifestyle advice	Pre-ablation	Not Specified
Ding et al. 2020	Retrospective matched cohort	UK	Intermittent Fasting	6 months pre-ablation	6 months
Donnellan et al. 2020	Retrospective matched cohort	US	Risk factor modification	Pre-ablation	Not Specified
Donnellan et al. 2019	Retrospective matched cohort	US	Bariatric surgery	Within 2 years pre-ablation	-
Jia et al. 2019	Prospective cohort	China	Self-initiation by patients	Post-ablation	12 months
Yaeger et al. 2019	Prospective cohort	US	Nurse-led weight loss clinic	12 months pre-ablation	12 months
Mohanty et al.2018	Prospective cohort	US	Diet and exercise plan	12 months pre-ablation	12 months
Pathak et al. 2014	Prospective cohort	Australia	Physician-led risk factor modification clinic	Post-ablation	24 months

*Conference Abstract, *US* United States, *UK* United Kingdom

### Recurrence of AF

**Fig. 1:**
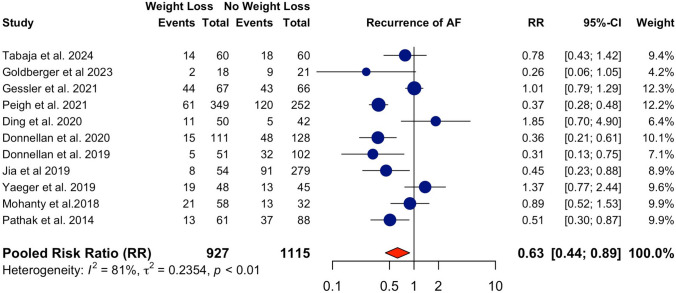
Forest plot for comparison of weight loss and non-weight loss groups. Successful achievement of weight loss, through whichever kind of intervention, was associated with a significant reduction in AF recurrence (RR: 0.63, 95% CI: 0.44–0.89, heterogeneity: I^2^ = 81% & τ.^2^ = 0.24)

A total of 13 studies were included in the analysis, grouped by intervention type to assess the impact on AF recurrence. The overall pooled risk ratio (RR) across all weight-loss interventions was 0.64 (95% CI: 0.47–0.87), indicating a statistically significant reduction in AF recurrence post-ablation (Graphical Abstract). There was high heterogeneity across studies (*I*^2^ = 86 & τ^2^ = 0.23). The differences between different types of interventions were non-significant (*p* = 0.28). Comparisons between weight loss and non-weight loss groups (after excluding two studies that did not mention weight change) showed that successful achievement of weight loss, through whichever kind of intervention, was associated with a significant reduction in AF recurrence (RR: 0.63, 95% CI: 0.44–0.89). Study heterogeneity remained high (*I*^2^ = 81% & τ^2^ = 0.24) (Fig. 1).

### GLP-1 receptor agonists

Four studies examined the effect of GLP-1 RA on AF recurrence post-ablation, resulting in a pooled RR of 0.63 (95% CI: 0.35–1.15). Overall, there was a trend toward reduced AF recurrence in the intervention group, though the result did not reach statistical significance. Satti et al. [11] reported no difference between intervention and control groups, while Tabaja et al. [12] and Goldberger et al. [13] observed a non-significant reduction. In contrast, Zou et al. [14] showed a significant reduction in AF recurrence with the perioperative use of GLP-1 RA. This subgroup demonstrated significant heterogeneity (*I*^2^ = 73% & τ^2^ = 0.24), indicating the outcome between studies varied. It should be acknowledged that although the study by Goldberger et al. used GLP-1 RA (liraglutide) for their patients, the exposure for comparison was pre-ablation weight loss of < 3% versus 3–10%, and therefore the exact attributable fraction owing to GLP-1 RA remained uncertain as of the time of this manuscript submission.

### Other interventions

Eight studies used LRFM as the weight-loss intervention, with a pooled RR of 0.68 (95% CI: 0.46–1.03). The analysis indicated a reduction in AF recurrence with LRFM to achieve weight loss, though the result was not statistically significant. This subgroup displayed a high heterogeneity (*I*^2^ = 86% & τ^2^ = 0.27)**,** suggesting high variability between studies**.**

Only one study investigated the effect of bariatric surgery on AF recurrence post-ablation, with a significantly reduced RR of 0.31 (95% CI: 0.13–0.75), indicating that bariatric surgery is associated with a significant reduction in AF recurrence compared to the control group.

### Timing and sustainability of weight loss interventions

**Fig. 2:**
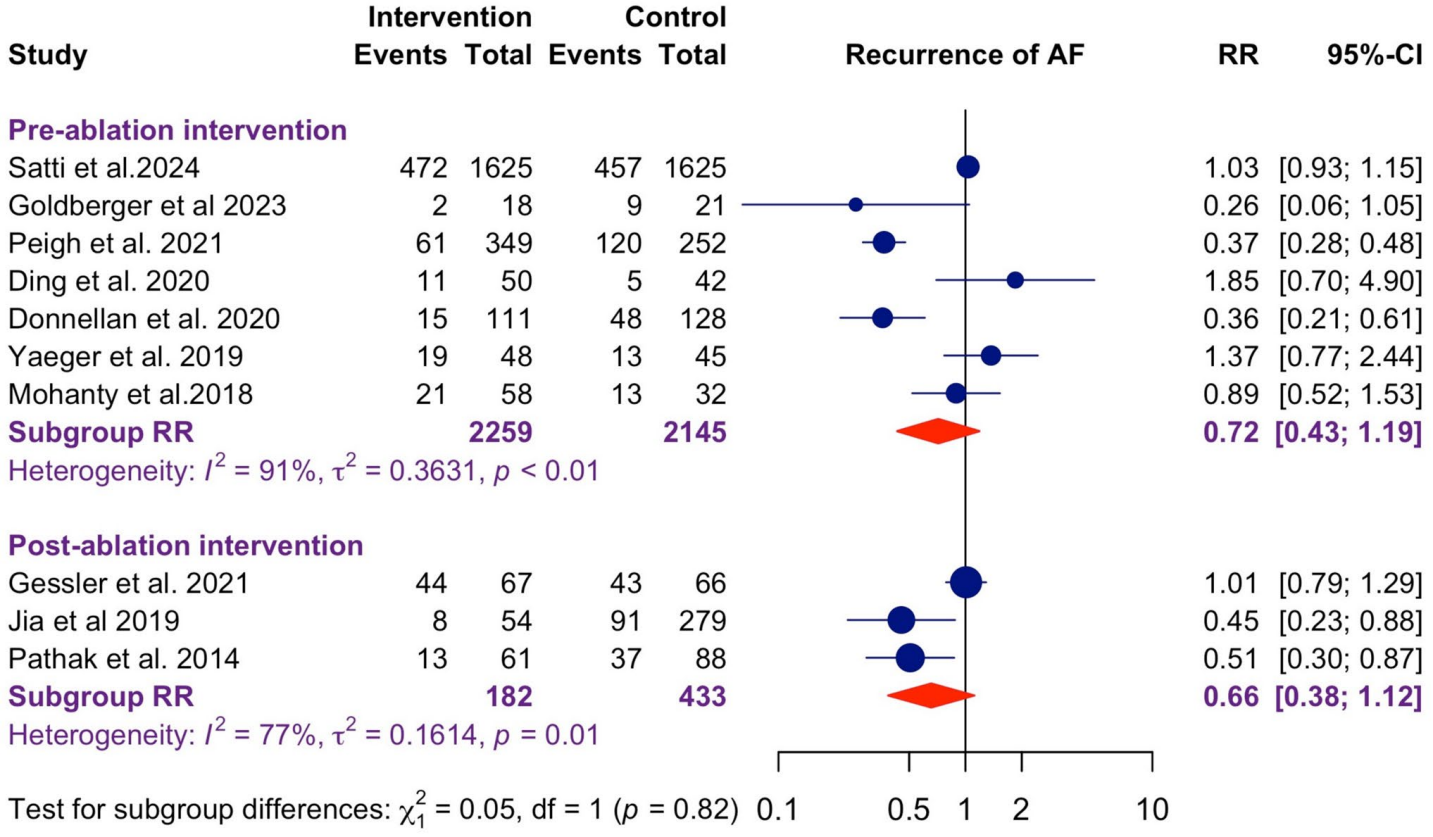
Forest plot for recurrence of AF stratified by initial time of intervention. Studies with weight loss interventions administered pre-ablation (pooled RR: 0.72, 95% CI: 0.43–1.19), had no significant difference versus those administered post-ablation (RR: 0.66, 95% CI: 0.38–1.12, between-groups *p* = 0.82)

The studies were stratified by the time of weight loss intervention, with the bariatric surgery study [23] excluded due to its one-time intervention nature and Zou et al. [14] excluded for unspecified intervention timing. This resulted in 10 studies for subgroup analysis on initiation timing of interventions, excluding Tabaja et al. [12], which involved patients starting on GLP-1 RA pre- and post-ablation (Fig. 2). Studies with pre-ablation interventions showed a pooled RR of 0.72 (95% CI: 0.43–1.19) for AF recurrence, while post-ablation interventions resulted in an RR of 0.66 (95% CI: 0.38–1.12), with no significant subgroup difference (*p* = 0.82), which suggested the initiation time of weight-loss interventions did not affect the ablation outcome.

**Fig. 3:**
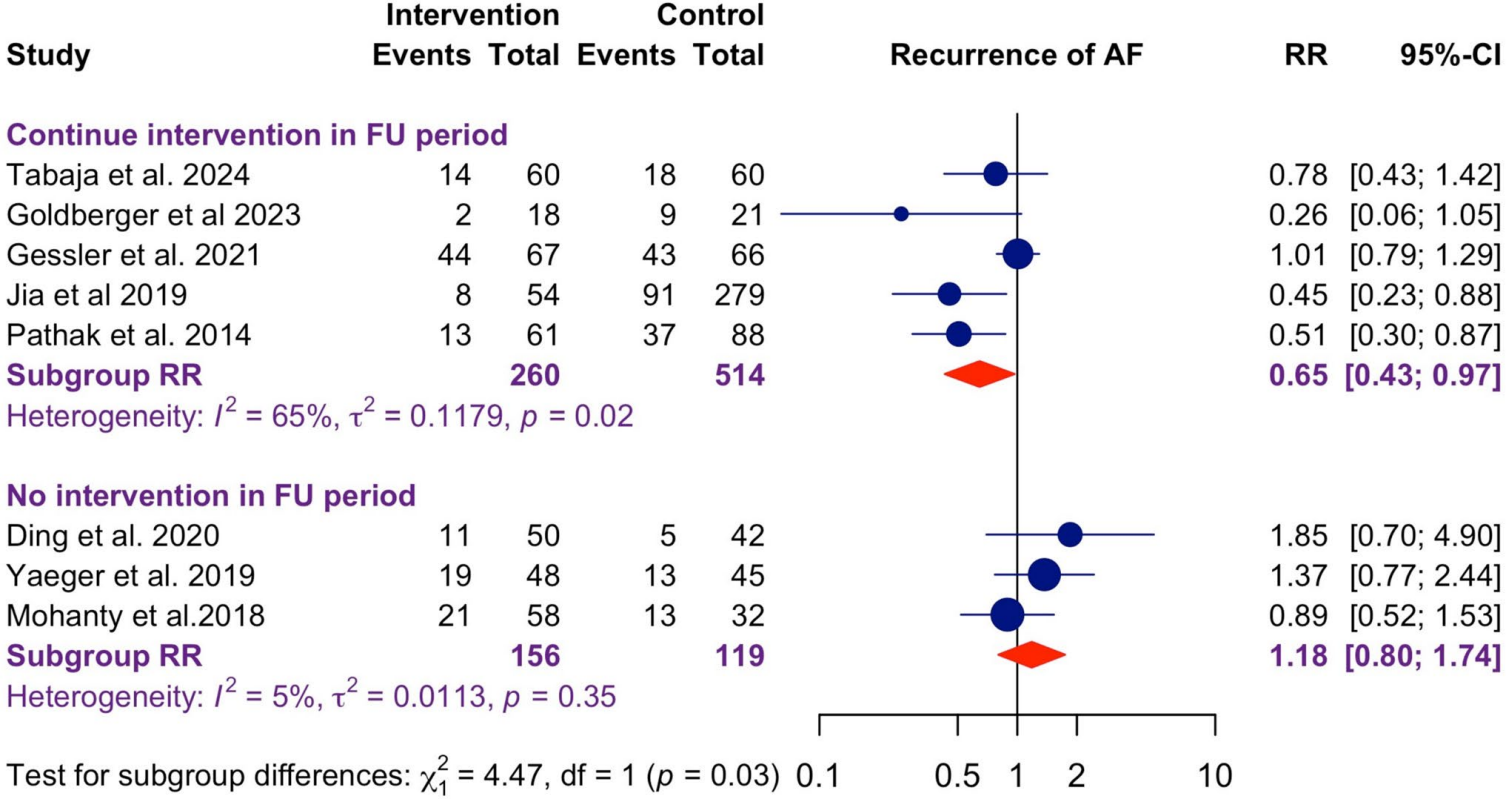
Forest plot for recurrence of af stratified by continuation of intervention beyond after ablation. Studies with sustained continuation of weight loss intervention post-ablation had a significant reduction in AF recurrence (RR 0.65, 95% CI: 0.43–0.97), but not those without sustained interventions (RR 1.18, 95% CI: 0.80–1.74), with significant between-groups difference (*p* = 0.03)

The studies were further divided based on the sustained continuation of weight loss intervention during the follow-up period. Eight studies were included after excluding three retrospective studies with unspecified completion times (Fig. 3). In studies with ongoing weight loss interventions during the follow-up period, a significant reduction in AF recurrence was observed (RR 0.65, 95% CI: 0.43–0.97). In contrast, studies without sustained interventions in the follow-up period showed no significant association with AF recurrence (RR 1.18, 95% CI: 0.80–1.74), with significant between-groups difference (*p* = 0.03). These suggest that sustained weight loss interventions in the follow-up period can improve ablation outcomes.

### Meta-regression

Meta-regression analyses were conducted to explore the influence of magnitude of weight loss and clinical characteristics on the study outcome.

**Fig. 4:**
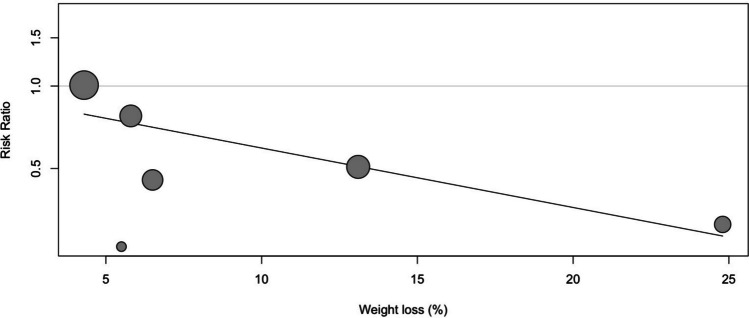
Meta-regression of magnitude of weight loss on the risk of recurrence of AF. For each 1% absolute decrease in body weight achieved, there was a 6% relative risk reduction in AF recurrence (RR 0.94, 95% CI: 0.90—0.99)

Importantly, the magnitude of weight loss, measured from baseline to the end of the follow-up period, significantly predicted risk of AF recurrence (RR 0.94, 95% CI: 0.90—0.99) (Fig. 4). This result also indicated that for each 1% absolute decrease in body weight achieved, there was a 6% relative risk reduction in AF recurrence. This effect was consistent regardless of the choice of weight loss intervention, and helped to account for the variance in effect size observed across studies.

**Fig. 5:**
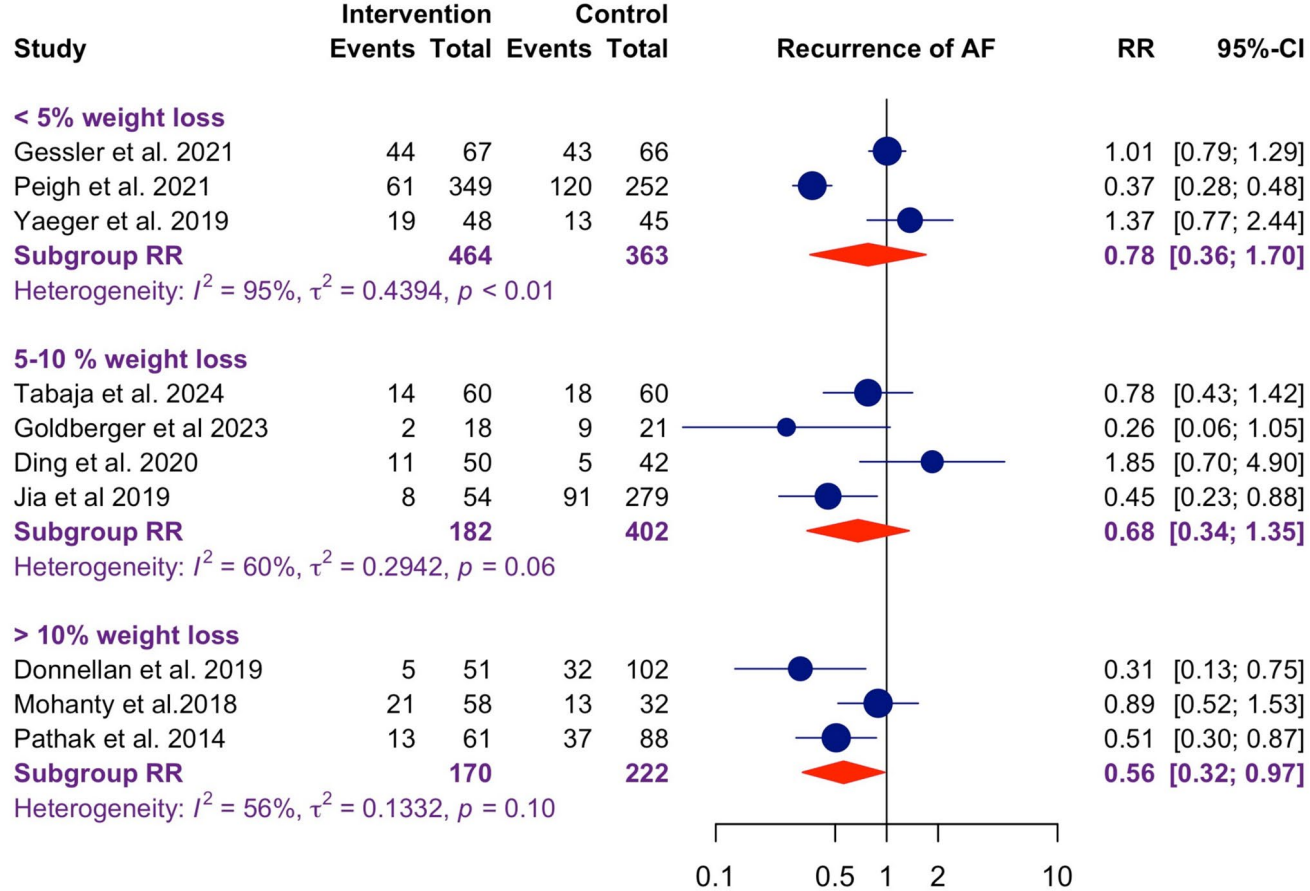
Forest plot for recurrence of AF stratified by magnitude of weight loss. Subgroup analyses for risk of AF recurrence for those achieving < 5%, 5–10%, and > 10% weight loss, were respectively RR: 0.78 (95% CI: 0.36–1.70), RR: 0.68 (95% CI: 0.34–1.35), and RR: 0.56 (95% CI: 0.32–0.97)

Based on the meta-regression, further sensitivity analyses were performed to further characterize the effect of magnitude of weight loss (Fig. 5). Unlike the meta-regression, which included six studies with detailed baseline and follow-up weight data, the subgroup analysis included 10 studies and stratified them by weight loss magnitude (< 5%, 5–10%, and > 10%). Results displayed a trend of progressively greater risk reduction in AF recurrence with increasing weight loss magnitude. The subgroup achieving > 10% weight loss demonstrated the most significant risk reduction (*RR* = 0.56, 95% CI: 0.32–0.97), and the 5–10% group also showed a favourable trend (*RR* = 0.68, 95% CI: 0.34–1.35), while the < 5% weight loss group had the least impact on reducing AF recurrence (*RR* = 0.78, 95% CI: 0.36–1.70).

In conjunction, other clinical characteristics including age, sex, AF type, comorbidities (e.g. hypertension, diabetes and obstructive sleep apnea), cardiac function by LVEF, baseline BMI and BMI at the end of follow-up were each assessed at separate meta-regression models. However, none of these variables showed a statistically significant association with the outcome.

### Sensitivity analysis

A leave-one-out analysis was done to assess the validity of the outcome. After taking out each study at a time, the pooled RR remained statistically significant, and the heterogeneity remained high (*I*^2^ = 75.5% to 87.6%), suggesting no significant influence from any single study. A funnel plot was drawn to assess publication bias, revealing no significant asymmetry, aligned with the Egger test with a *p*-value of 0.0848, suggesting no significant evidence of publication bias. Sensitivity analysis was also done by excluding the three conference abstracts, which resulted in a statistically significant pooled RR of 0.68 (95% CI: 0.48–0.98), suggesting the inclusion of these three conference abstracts was justified and supported the findings regarding the effectiveness of weight-loss interventions in reducing AF recurrence.

## Discussion

### Effects of weight loss on AF recurrence

To our knowledge, this meta-analysis is the first to comprehensively evaluate the effects of different weight loss interventions on the recurrence of AF after catheter ablation in obese patients. Our findings demonstrate a significant reduction in AF recurrence, with a pooled RR of 0.64 (95% CI: 0.47–0.87) among those who underwent weight-loss interventions. Obesity is known to increase blood volume and cardiac output, leading to left atrial enlargement, left ventricular hypertrophy and elevated pulmonary pressures, thus increasing the risk of AF occurrence [24–26]. Additionally, metabolic changes associated with obesity, such as insulin resistance, hypertension, and activation of the renin–angiotensin–aldosterone system (RAAS), contribute to atrial remodelling, further elevating the risk of AF [24, 25]. Obesity-related conditions, such as obstructive sleep apnoea, also increase AF susceptibility by causing recurrent cycles of hypoxia, inflammation, and abnormal autonomic responses [27]. Another contributing factor is epicardial adipose tissue in obese individuals, which releases inflammatory mediators and adipokines, which can lead to myocardial fibrosis and electrical disturbances, triggering AF through paracrine signalling pathways [28–31].

In obese AF patients, weight loss has been shown to significantly reduce AF incidence and severity [4, 18, 32] through the direct effects on cardiac structure and function and indirect effects from the correction of other cardiovascular risk factors [24, 25]. Weight reduction in obese patients has also been associated with decreased left atrial volume and left ventricular wall thickness, thus reducing AF risk [4, 18, 33]. Previous meta-analyses found that achieving sustained weight loss, particularly > 10%, significantly reduced AF recurrence after ablation [34, 35]. We also found that greater weight loss had better ablation outcomes in the meta-regression and subgroup analysis. Our findings along with these studies, highlights the importance of weight management as an essential perioperative care for obese AF patients.

### Comparisons of GLP-1 RA versus other weight loss interventions

From our study, GLP-1 RA and LRFM each showed a trend towards reduced AF recurrence, but did not reach statistical significance. In comparison, bariatric surgery showed a significant reduction in AF recurrence post-ablation. Bariatric surgery is well-recognized for sustainable and long-term weight loss [36]. Previous studies supported the potential benefit of bariatric surgery in AF prevention among morbidly obese patients. A significant reduction in AF incidence was found among patients undergoing bariatric surgery compared to those receiving other medical treatment [37, 38]. These findings suggested a long-term benefit of bariatric surgery in lowering AF risk in obese patients. Donnellan et al. [23] further showed the additional advantage of bariatric surgery for patients undergoing AF ablation procedures by improving ablation outcome through effective and sustainable weight management.

GLP-1 RA may prevent AF by reducing atrial remodelling [39] through enhanced myocardial metabolism, stabilised blood glucose, and regulating the RAAS in animal studies [40, 41]. However, human studies have shown inconclusive results regarding GLP-1 RA’s clinical efficacy in preventing AF. Prior meta-analyses of RCTs comparing GLP-1 RA to placebo in obese patients showed statistically non-significant reductions in the occurrence of AF by [42, 43]. In contrast, a meta-analysis of 6,031 individuals found a significant reduction in AF recurrence at 12 months post-ablation among obese patients using GLP-1 RA (Hazard Ratio 0.549, 95% CI: 0.315- 0.956) [44]. Additionally, several meta-analyses of RCTs have explored the effectiveness of GLP-1 RA in reducing AF occurrence in patients with type 2 diabetes, comparing GLP-1 RA against placebo or other hypoglycaemic agents. Among these, two studies [45, 46] reported significant reductions in AF occurrence with the use of GLP1 RA, while three [47–49] observed non-significant reductions. The lack of statistical significance or borderline statistical significance from the studies by us and some others may therefore potentially indicate that study samples included for this specific clinical setting was under-powered. Our study indicated that once weight loss was achieved, be it by GLP-1 RA or other weight loss intervention, it reduced the risk of AF recurrence.

### Importance of sustained continued weight control following AF ablation

Earlier studies showed conflicting results in terms of the effectiveness of LRFM in improving AF ablation outcomes, with both positive [18, 21–23] and negative results [16, 17, 19, 20]. Our study found that the sustained continuation of weight loss post-ablation is an critical determining factor of the ultimate effectiveness any weight loss program. This aligns with the landmark study LEGACY, in which weight fluctuation in obese patients led to a higher risk of AF recurrence compared to those with sustainable weight loss, as greater stability in weight management was associated with improved cardiac remodelling and reduced AF recurrence [4].

Regarding the optimal time for weight loss interventions, both pre-ablation and post-ablation weight loss interventions showed similar outcomes in reducing AF recurrence. However, obesity significantly impacts respiratory mechanics, increasing the risk of perioperative anaesthesia complications [50]. Therefore, pre-ablation weight loss may offer additional benefits by reducing these risks.

### Clinical implications

This meta-analysis highlights the importance of perioperative weight loss interventions for obese AF patients who undergo AF ablation, as weight management reduces AF recurrence post-ablation. Weight loss programs with long-term support to prevent weight regain should be provided as part of perioperative care for obese AF patients to improve ablation outcomes.

Lifestyle risk factor modifications are always recommended for AF patients, especially for those with obesity and high-risk factors [1]. However, achieving and maintaining weight loss through lifestyle and risk factor changes alone can be challenging. Long-term follow-up and close monitoring are essential to support adherence to the weight loss plan and achieve sustainable weight loss. For patients requiring additional support, both surgical and pharmacological weight loss methods can supplement LRFM to achieve and maintain weight loss goals.

Bariatric surgery showed significant reductions in AF recurrence post-ablation, likely due to its ability to sustain weight loss more effectively than LRFM alone. However, bariatric surgery is generally indicated only for patients with a BMI over 35 kg/m^2^ or a BMI over 30 kg/m^2^ with metabolic disease. Due to the potential risks, bariatric surgery may not be suitable for frail patients or those with multiple comorbidities [51].

For patients who are not suitable or prefer not to undergo surgery, GLP-1 RA offers a non-invasive alternative by reducing appetite and caloric intake to support weight loss maintenance with a healthy lifestyle. Additionally, GLP-1 may potentially provide direct cardiac therapeutic benefits, although further clinical data is needed. The choice of GLP-1 RA type, dosage, and duration will need careful consideration, as weight regain has been observed following the cessation of GLP-1 RA therapy [52]. Different specific types of GLP-1 RA may also affect weight loss outcome [5]. Managing potential adverse reactions, including gastrointestinal side effects, is essential.

In all cases, whether with pharmacological or surgical interventions, maintaining a healthy lifestyle and adjustment of cardiovascular risk factors remain crucial for overall cardiovascular health. Sustainability is the key in any weight loss program to reduce AF recurrence.

### Limitations

This meta-analysis has several limitations. The heterogeneity across studies was high, which may be due to the variation in design, populations, intervention protocols, and definitions of AF recurrence, which limit the generalizability of our study. The definition of AF recurrence varied widely across studies, while several studies defined AF recurrence as atrial arrhythmia episodes ≥ 30 s, one study relied on clinical indicators like the need for AF-related treatments and some did not specify criteria. Additionally, two studies included AF episodes occurring within the blanking period (the initial 3 months post-ablation), which may overestimate the recurrence rates. Moreover, most included studies were observational, and though the majority were propensity score matched, this still increased the potential for bias and confounding. More than half of the included studies were retrospective, which limited the explanatory power due to missing data.

Data-related limitations also impacted the analysis. Adherence to weight loss interventions was inconsistently reported between studies, with some including all patients in the intervention group, regardless of the adherence, while some studies only included participants who strictly adhered to the weight loss strategies and achieved weight loss. This discrepancy limits the comparability and generalizability of the findings. The inclusion of conference abstracts also limits the completeness and quality of data, as these studies lacked details. Subgroup analyses were constrained by the limited number of studies available. Specifically, only one study examined the effect of bariatric surgery, thus limiting statistical power. This is a study limitation that ought to be acknowledged, and findings should be interpreted with caution based on a single study. Furthermore, in two studies (Yaeger et al. [19] and Ding et al. [20]), weight loss interventions were showed to demonstrate a rather opposing trend of being associated with a higher risk of AF recurrence post-ablation, albeit there were not statistically significant. The findings are is not expected, and such discrepancies compared to our overall results warrant to be further explored in future studies. Lastly, there were only six studies included in the meta-regression analysis of the magnitude of weight loss, which may reduce the ability to detect true effects and increase the risk of overfitting. However, this result was consistent with findings in the subgroup analysis and previous studies [34, 35]. This consistency suggests a relationship between weight loss and AF recurrence and highlights the potential of weight loss magnitude as an important factor in explaining the high heterogeneity between studies.

## Conclusions

Peri-operative weight loss interventions including GLP-1 RA in obesity patients, significantly reduced AF recurrence after catheter ablation, in a dose–response manner and irrespective of the specific choice of intervention strategy. Sustained continuation of weight loss post-ablation is a key determinant of success.

## Supplementary Information

Below is the link to the electronic supplementary material.

Supplementary file1 (PPTX 143 KB)

Supplementary file2 (DOCX 25 KB)

## Data Availability

Data presented in this study will be made available to researchers upon reasonable request to the corresponding author.
