# Supplementary figures and images for: Comparing GLP-1 agonists versus other weight loss interventions on risk of atrial fibrillation recurrence after catheter ablation: a meta-analysis
Source: J Interv Card Electrophysiol. 2026 Mar 17;69(4):753–64. doi: 10.1007/s10840-026-02297-8 (PMC13221330; doi:10.1007/s10840-026-02297-8)

## Slide 1
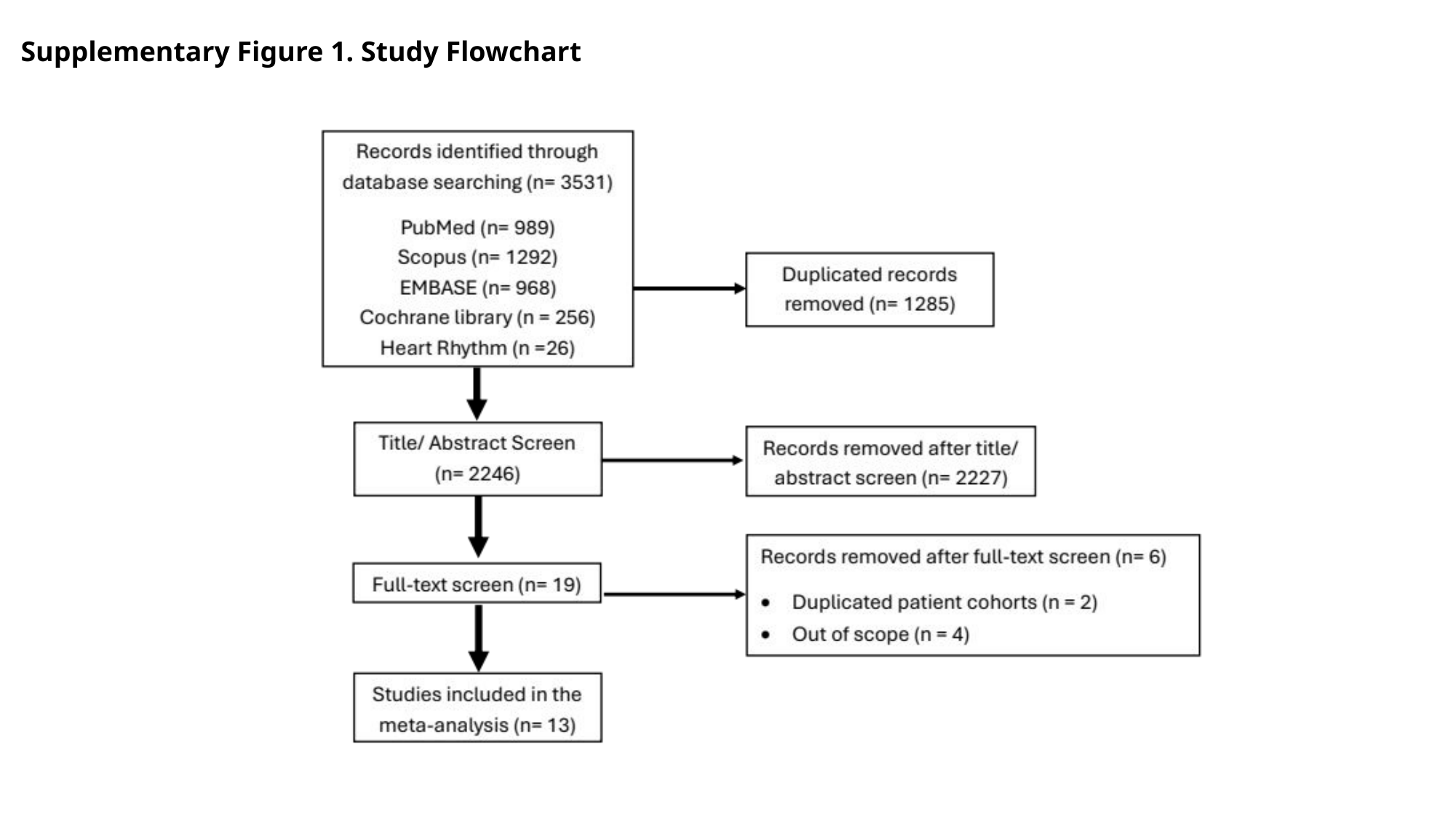

Supplementary Figure 1. Study Flowchart

Supplement: Supplementary file 1 — Supplementary file1 (PPTX 143 KB) [file 10840_2026_2297_MOESM1_ESM.pptx]
