# Supplementary material for: Comparing GLP-1 agonists versus other weight loss interventions on risk of atrial fibrillation recurrence after catheter ablation: a meta-analysis
Source: J Interv Card Electrophysiol. 2026 Mar 17;69(4):753–64. doi: 10.1007/s10840-026-02297-8 (PMC13221330; doi:10.1007/s10840-026-02297-8)
Supplement: Supplementary file 2 — Supplementary file2 (DOCX 25 KB) [file 10840_2026_2297_MOESM2_ESM.docx]

Supplementary Table 1. Baseline characteristics

| **Study** |  | **Sample (n)** | **Mean Age ± SD** | **Female** | **PAF** | **HT** | **DM** | **OSA** | **LVEF** | **Mean Baseline BMI (kg/m^2^)** | **Mean BMI at the end of follow-up (kg/m^2^)** | **Weight change** |
| --- | --- | --- | --- | --- | --- | --- | --- | --- | --- | --- | --- | --- |
| **Satti et al.2024** | Intervention | 1625 | 63.7 ± 8.8 | 33% | 67% | 76% | 75% | - | - | - | - | - |
|  | Control | 1625 | 63.9 ± 10.3 | 34% | 68% | 77% | 79% |  |  |  |  |  |
| **Tabaja et al. 2024** | Intervention | 60 | 65 ± 5 | 27% | 35% | - | - | - | - | 36 | 33.9 | -5.8% |
|  | Control | 60 |  |  |  |  |  |  |  | 118kg | 116kg | -1.7% |
| **Zou et al. 2024** | Intervention | 61 | - | - | - | - | - | - | - | - | - | - |
|  | Control | 62 |  |  |  |  |  |  |  |  |  |  |
| **Goldberger et al 2023** | Intervention | 18 | 62 ± 9 | 27% | 20% | 85% | 27% | 44% | 56% | 36.1 | 34.1 | -5.6% |
|  | Control | 21 |  |  |  |  |  |  |  |  | 36.8 | +0.2% |
| **Gessler et al. 2021** | Intervention | 67 | 58.7 ± 11.6 | 46% | 33% | 81% | 13% | 32% | 57% | 34.9 | 33.4 | -1.5% |
|  | Control | 66 | 62.1 ± 9.1 | 48% | 52% | 88% | 21% | 41% | 60% | 34.8 | 34.5 | -0.9% |
| **Peigh et al. 2021** | Intervention | 349 | 62.1 ± 10.3 | 32% | 52% | 48% | 15% | 17% | 56% | 29.5 | - | - |
|  | Control | 252 |  |  |  |  |  |  |  |  |  |  |
| **Ding et al. 2020** | Intervention | 50 | 64.9 ± 9.5 | 50% | 38% | - | - | - | - | 36.5 | - | - |
|  | Control | 42 | 64.1 ± 8.7 | 36% | 50% |  |  |  |  | 35.3 |  |  |
| **Donnellan et al. 2020** | Intervention | 111 | 66 ± 9 | 42% | 29% | 82% | 33% | 73% | 57% | 40.8 | - | - |
|  | Control | 128 |  |  |  |  |  |  |  |  |  |  |
| **Donnellan et al. 2019** | Intervention | 51 | 63.4 ± 8.8 | 49% | 39% | - | - | - | - | 47.6 | 35.8 | -24.8% |
|  | Control | 102 | 63.4 ± 9 | 49% | 23% |  |  |  |  | - | - | - |
| **Jia et al 2019** | Intervention | 54 | - | 28% | 59% | 63% | 20% | - | - | - | - | -1.77 kg/m^2^ |
|  | Control | 279 |  | 27% | 57% | 63% | 24% |  |  |  |  | 0 |
| **Yaeger et al. 2019** | Intervention | 48 | - | - | - | - | - | - | - | - | - | - |
|  | Control | 45 |  |  |  |  |  |  |  |  |  |  |
| **Mohanty et al.2018** | Intervention | 58 | 62 ± 9 | 29% | 0% | 59% | 19% | 22% | 54% | 38 | - | - |
|  | Control | 32 | 64 ± 10 | 25% | 0% | 63% | 22% | 25% | 55% | 37 |  |  |
| **Pathak et al. 2014** | Intervention | 61 | 58.4 ± 10.8 | 44% | 65% | 87% | 15% | - | 61% | 33.5 | 29.1 | -13.1% |
|  | Control | 88 | 57.2 ± 9.9 | 31% | 56% | 83% | 19% |  | 60% | 32.1 | 31.8 | - 0.9% |

SD: Standard deviation; PAF: Paroxysmal Atrial Fibrillation; HT: Hypertension; DM: Diabetes Mellitus; OSA: Obstructive Sleep Apnea; LVEF: Left ventricular ejection fraction; BMI: Body Mass Index

Supplementary Table 2. Definition of AF Recurrence

| **Study** | **Criteria** | **Time After Ablation** |
| --- | --- | --- |
| Satti et al.2024 | Not require Cardioversion No new initiation of class I/III AAD therapy, nor repeat AF ablation | 3-12 months |
| Tabaja et al. 2024* | Not specified | 3-12 months |
| Zou et al. 2024* | Not specified | 0-12 months |
| Goldberger et al 2023* | Not specified | 3-6 months |
| Gessler et al. 2021 | AF ≥ 30 seconds | 3-12 months |
| Peigh et al. 2021 | Documented atrial arrhythmia >30 seconds | 3-15 months |
| Ding et al. 2020 | Not specified | 0-12 months |
| Donnellan et al. 2020 | Documented atrial arrhythmia ≥ 30 seconds | 3-10 months |
| Donnellan et al. 2019 | Documented atrial arrhythmia ≥ 30 seconds | 3-12 months |
| Jia et al 2019 | Documented atrial arrhythmia ≥ 30 seconds | 3-12 months |
| Yaeger et al. 2019 | Atrial arrhythmia ≥ 30 seconds | At 12-month |
| Mohanty et al.2018 | Not specified | 3-12 months |
| Pathak et al. 2014 | Atrial arrhythmia ≥ 30 seconds | 3-12 months |

AAD: Antiarrhythmic drug; AF: Atrial Fibrillation
